# Veterinary Medical Association of New Jersey

**Published:** 1895-01

**Authors:** S. Lockwood

**Affiliations:** Secretary


Secretary.

### VETERINARY MEDICAL ASSOCIATION OF NEW JERSEY.

The thirty-third regular meeting of the Veterinary Medical Association of New Jersey was held at the State Street House, Trenton, December 13, 1894.

Meeting called to order at 12.05 o'clock. Nine members present and one delegate from the Pennsylvania Association, Dr. Thos. B. Rayner, of Chestnut Hill, Philadelphia, Pa. Minutes of previous meeting read and approved. The President's address was in the form of a report of veterinary practice as at present carried on in this State. In some counties there were more illegal practitioners than legal. As he had not finished the census of the whole State, he did not give any names, but would try and have the full list ready for the next meeting.

The Treasurer reported $35.74 in the treasury and all bills paid. Committee on Revision of By-laws reported the changes made, and the Secretary was ordered to report the same to Board of Trustees in writing. Delegates to sister Associations all had excuses.

Dr. Gerth being the only essayist present, read a paper on "Horse Meat as an Article of Food."

Meeting adjourned at 1 P.M. for dinner.

Meeting reconvened at 2.30. After the reading of several letters the discussion of Dr. Gerth's paper was resumed.

The Secretary was requested to read a copy of a bill proposed to be introduced in the Pennsylvania Legislature the coming session for the appointment of a State Cattle Commission. It was a well-drafted bill, and should become a law in every State in the Union.

Drs. Miller and Dustan reported the steps taken by them in reference to their names being used as reference on a patent medicine circular. Their report was accepted, and they were exonerated from further blame.

Meeting adjourned at 4.10 P.M. to meet at the same place in April.

S. LOCKWOOD,
Secretary.

